# Optimal size and PEG coating of gold nanoparticles for prolonged blood circulation: a statistical analysis of published data†

**DOI:** 10.1039/d4na00782d

**Published:** 2025-01-13

**Authors:** Dmitry Nevozhay, Ronald Rauch, Zhongya Wang, Konstantin V. Sokolov

**Affiliations:** a Department of Imaging Physics, The University of Texas MD Anderson Cancer Center Houston TX 77030 USA dnevozhay@mdanderson.org ksokolov@mdanderson.org; b Department of Biostatistics, The University of Texas MD Anderson Cancer Center Houston TX 77030 USA; c Department of Bioengineering, Rice University Houston TX 77005 USA; d Department of Biomedical Engineering, The University of Texas at Austin Austin TX 78712 USA

## Abstract

This study presents a statistical analysis of how gold nanoparticle (GNP) size and polyethylene glycol (PEG) coating molecular weight (MW) affect the circulation of nanoparticles in blood. We showed a non-linear relationship with interaction between GNP size and PEG MW. The findings revealed a threshold effect, and recommendations for GNP coating are discussed.

## Abstract 2

A statistical meta-analysis of gold nanoparticles reveals non-linear interactions. The optimal design (diameter ≤ 40 nm and PEG ≥ 5 kDa) maximizes blood circulation, providing evidence-based guidelines for enhanced nanoparticle design in biomedicine.

## Graphical abstract

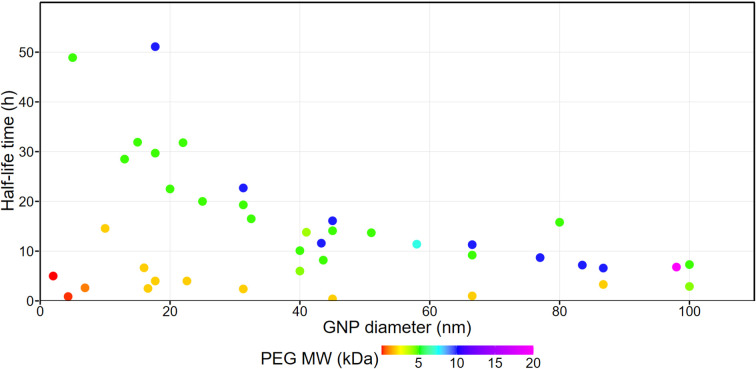

## Introduction

Gold nanoparticles (GNPs) are an important platform for various biomedical applications, ranging from diagnostics to drug delivery.^1–10^ The robust synthesis methodology, functionalization potential, and well-studied physical and chemical properties of GNPs contributed to their successes, particularly in *in vitro* diagnostics, especially lateral flow assays.^11^ Specific applications include diagnostic tests for pregnancy,^12,13^ infectious diseases,^14,15^ and other^16,17^ diseases. However, their applications in therapy and drug delivery are rather limited, partly due to existing challenges with pharmacokinetics (PK) and biodistribution.^18,19^

The size of GNPs is a critical factor in determining their clearance from the blood and biodistribution inside the body.^20^ It was shown that for particles with sizes above the renal threshold (*i.e.*, ∼5 nm in mice),^21^ the half-life of circulation in the blood generally increases as the size decreases.^22–27^ In addition, coating the GNPs with PEG molecules of various MWs has long been established as an effective method to prolong nanoparticles' circulation in blood.^26,28,29^ This process, known as PEGylation, reduces opsonization and subsequent uptake by the reticuloendothelial system (RES).^29–31^ While both the size of GNPs and the characteristics of the PEGylation process were investigated for their impact on the PK of GNPs, considerable variability exists across published studies. Because of this, there remains a lack of comprehensive understanding regarding how the size of GNPs and the molecular weight of PEG coating interplay to influence nanoparticles' half-life in the bloodstream. This meta-analysis paper aims to address this knowledge gap by systematically analysing data from published studies. The primary focus is to elucidate the dependency of the blood half-life time of GNPs on their size and PEG MW. Using published sources and rigorous statistical analysis, we were able to outline practical GNP design recommendations for maximizing their circulation in blood. We also outlined the directions for further evaluation of these factors and provide a ready-to-use statistical framework for additional analysis once more published data become available.

## Results and discussion

### Visual assessment

**Fig. 1:**
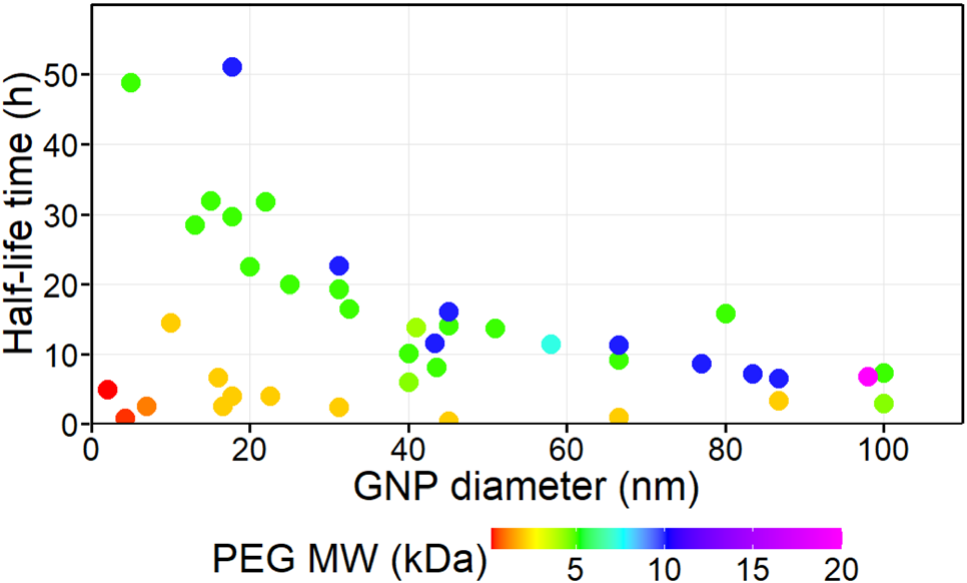
Data points selected for statistical analysis. The colour of the data point corresponds to the MW of the PEG coating (scale below).

We conducted initial visual assessment of the data to identify patterns and potential outliers. The diameter of GNPs ranged from 2 to 100 nm and the MW of PEG ranged from 0.2 to 20 kDa. The scatter plots of half-life against GNP size and PEG size indicated a non-linear relationship (Fig. 1). Furthermore, visual assessment suggested the presence of two distinct trends in the data, divided by the MW of PEG, specifically (i) a primary trend for GNPs coated with higher MW PEG (≥5 kDa) displaying an inverse and distinctly non-linear relationship between GNP size and half-life time in blood; (ii) a secondary trend for GNPs coated with lower MW PEG (≤2 kDa) consistently displaying low half-life time values, irrespective of the GNP size.

### Statistical method and model selection

Considering the non-linear relationship between GNP size, PEG MW, and half-life time, especially in light of potential interaction between GNP size and PEG MW, we decided to use the Generalized Additive Model (GAM) to probe the statistical relationship among these variables as the GAM does not assume a linear relationship between variables and is generally well suited for capturing complex non-linear relationships.^32^

Assessing the distribution of the half-life variable, we found it to be highly skewed to higher values (Fig. S1A†), prompting a square root transformation to better approximate a normal distribution (Fig. S1B†). To mitigate overfitting, we applied the Restricted Maximum Likelihood (REML) method for selecting smoothing parameters instead of the default minimized generalized cross-validation (GCV) method used in the GAM function. Employing REML was suggested to yield more reliable and stable results.^32^ We fit two statistical models using the GAM function, with one dependent variable – half-life time and two independent variables – GNP size and PEG MW. Model 1 assumed no interaction between GNP size and PEG MW, while Model 2 incorporated an interaction term (R code for statistical analysis can be found in the ESI†).

Both models demonstrated statistically significant main effects (*p* < 0.001). However, Model 2 with an interaction term exhibited better performance (adjusted *R*^2^ = 0.90 and deviance explained = 92.3%) in comparison to Model 1 (adjusted *R*^2^ = 0.82, deviance explained = 85.1%). In addition, the improvement in explanatory power of Model 2 with interaction was also supported by its lower REML score (43.0) in comparison to Model 1 (REML score = 50.1). Moreover, ANOVA comparisons confirmed a statistically significant enhancement in the explanatory power of Model 2 over Model 1 (*p* < 0.001). In addition, Model 2 showed a lower Akaike Information Criterion (AIC) and Bayesian Information Criterion (BIC), indicating better model fitting in Model 2 despite its increased complexity.^33^Table 1 summarizes performance metrics of both models.

**Table 1:** Performance metrics of Model 1 (no interaction) and Model 2 (with interaction)

Model	Adjusted *R*^2^	Deviance explained, %	REML score	AIC	BIC
1 (no interaction term)	0.82	85.1	50.1	92.9	109.4
2 (with interaction term)	0.90	92.3	43.0	71.3	92.4

Next, we performed the diagnostic analyses for both models to assess their validity (Fig. S2 and S3†). Specifically, Q–Q plots, assessing how well model residuals align with a normal distribution, displayed patterns close to a straight line (Fig. S2A and S3A†). For Model 2, its residuals were distributed around 0 in residual plots, with a tighter clustering along the diagonal line in (Fig. S2B and S3B†) the plot of response against fitted values (Fig. S2D and S3D†). In addition, the histogram of residuals from Model 2 displayed a more symmetrical bell-shaped distribution (Fig. S2C and S3C†). All these findings show that Model 2, which includes the interaction term, was more efficient in explaining the relationship between GNP size, PEG MW and the half-life time in blood.

### Interpretation of statistical model findings

**Fig. 2:**
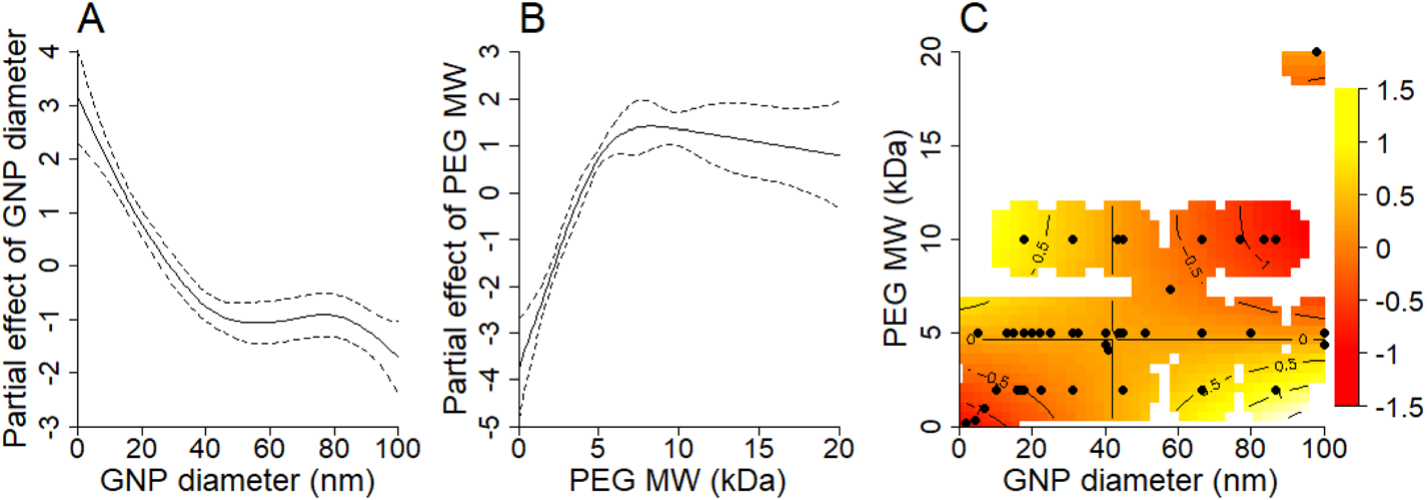
Main effects (A) of GNP diameter and (B) PEG MW; (C) Interaction term, representing how the combined effects of GNP size and PEG MW differ from what would be expected if these factors acted independently. Regions in yellow colour indicate a positive interaction effect while red colour indicates a negative interaction effect. Dashed lines represent confidence intervals in A and B and dark dots represent data points in C.

Upon choosing Model 2, we plotted *y* axis with the estimated main effects of GNP size (Fig. 2A), PEG MW (Fig. 2B), and the interaction effect between both (Fig. 2C) with *x*-axis representing the related GNP size, PEG MW, and the interaction, respectively. Decreasing GNP size below 40 nm has a beneficial effect on prolonging their half-life time in blood after the administration (Fig. 2A). Additionally, for GNPs larger than 40 nm, there appears to be no clear dependency between GNP size and half-life time in blood. Increasing PEG MW to 5 kDa has a beneficial effect on prolonging the half-life time of GNPs in blood (Fig. 2B), but PEG with sizes above 5 kDa does not have any additional benefit.

Interpretation of the GAM's interaction term is more nuanced and cannot be done without considering the main effects as well. The interaction term in Fig. 2C represents how the combined effects of GNP size and PEG MW differ from what would be expected if these factors acted independently. The heat map shows regions where this interaction is either positive (yellow) or negative (red), with the intensity indicating the strength of the interaction effect. We can see two regions which show positive interaction terms. The first yellow region, characterized by GNP size < 40 nm and PEG MW > 5 kDa (top-left yellow area in Fig. 2C), shows a strong positive interaction, indicating that these parameter combinations result in circulation times significantly longer than those predicted by the individual main effects alone. This synergistic effect suggests that small GNPs coated with high-MW PEG result in optimal properties for extended circulation. The bottom-right yellow region in Fig. 2C (bottom-right; GNP size > 60 nm and PEG MW < 5 kDa) also shows a positive interaction, but its practical significance is limited because it is overshadowed by the negative main effects of both large GNP size and low PEG MW (as shown in Fig. 2A and B). This illustrates how interaction effects must be interpreted in conjunction with main effects to understand their practical implications.

The interaction model's findings underscore the complex interplay between GNP size and PEG MW in determining the half-life of PEG-coated GNPs in the bloodstream. This statistical analysis highlighted a critical finding: coating GNPs with PEG which has a MW of 2 kDa or lower has minimal impact on prolonging the half-life time of GNPs in blood across all GNP sizes, whereas PEG molecules of 2 kDa or larger can significantly extend the half-life time of GNPs in blood, particularly smaller GNPs (less than 40 nm). The threshold effect observed for PEG suggests a minimal MW necessary for the effective extension of GNP circulation time. It is also worth mentioning that we had only three data points (ranging from 4.1 to 4.4 kDa) for PEG MW between 2 and 5 kDa (Table S1 in the ESI†). These sparse data make pinpointing the exact “watershed” PEG MW more challenging. The model suggests that a PEG MW of ≤2 kDa doesn't significantly enhance half-life, while a PEG MW of ≥5 kDa is already sufficient. Pinning down the minimum PEG MW that offers tangible benefits for extending the half-life time of GNPs could be a compelling direction for future studies.

While numerous factors influence nanoparticle circulation,^26,29,34,35^ including shape, surface charge, rigidity, PEG branching^36,37^ and grafting density, our analysis focused on GNP size and PEG MW due to the availability of sufficient quantitative data across multiple studies. The high explanatory power of our model (*R*^2^ = 0.90 and deviance explained = 92.3%) provides statistical evidence supporting previous observations that these parameters are key determinants of blood circulation time.^26,29^

### Limitations of the study

27 out of 41 data points are derived from two published studies (Table S1†). However, visual inspection of the dataset does not show any evidence of systematic bias based on the source of the data points (Fig. S4†). In addition, there was a relevant study^38^ that assessed the biodistribution of PEGylated GNPs in mice, but it did not report numerical values for blood half-life times and therefore could not be included in this analysis. However, it is important to mention that data from that report qualitatively support the conclusions derived from our statistical model. Specifically, GNPs with a diameter of 10 nm coated with 5 kDa PEG exhibited a higher concentration in the mouse blood 24 hours after systemic injection compared to similarly sized GNPs coated with 2 kDa PEG.^38^

As can be seen from Fig. 2C, the model's predictive power diminishes for PEG MW > 10 kDa because there was only one data point for PEG MW 20 kDa (Table S1†). In addition, we already mentioned the lack of granularity in establishing the exact PEG MW threshold between 2 and 5 kDa, which could be an interesting direction for follow up experimental studies. We provide the full R code in the ESI† so once additional experimental data become available, they can be directly plugged into this analysis to refine the statistical model.

Our statistical analysis is based on 41 published data points, with 5 kDa PEG coating representing 39% of all values. While we acknowledge a modest sample size, several statistical indicators support the robustness of our conclusions. The model demonstrates high explanatory power (adjusted *R*^2^ = 0.90 and deviance explained = 92.3%) with highly significant effects (*p* < 0.001), and diagnostic plots show good model fit and residual behaviour (Fig. S2 and S3†). To address potential concerns about sample size and data imbalance, we performed two additional analyses. First, the weighted GAM analysis produced results consistent with our original model (Fig. S5†), suggesting that the overrepresentation of 5 kDa PEG data points does not significantly affect our conclusions. Second, sensitivity analysis using bootstrapped datasets also confirmed the validity of our findings (Fig. S6†). The robustness of results across different analytical approaches, combined with strong model performance metrics, provides confidence in our findings despite dataset limitations.

Moreover, conducting a meta-analysis inherently involves balancing statistical complexity and interpretability. We addressed this by applying a GAM methodology, which captures non-linear relationships while controlling for overfitting through REML optimization. However, we acknowledge that this approach, while powerful and allowing for focused statistical analysis, simplifies a complex biological system to a certain degree. Our focus on GNP size and PEG MW allowed us to establish practical design guidelines, but other factors likely contribute to the observed variations in circulation times.

Finally, meta-analyses also face unique challenges, including data heterogeneity and variability in experimental protocols, incomplete reporting, and potential publication bias. Future meta-analyses would benefit from standardized reporting of nanoparticle characterization data, detailed experimental protocols, raw pharmacokinetic measurements. Adopting more sophisticated statistical approaches, like Bayesian hierarchical models and machine learning algorithms may enable handling sparse, multidimensional datasets with potentially missing values while maintaining interpretability.

## Conclusions

The findings reported here provide a practical recommendation: using GNPs ≤ 40 nm in diameter and coating them with PEG with a MW of at least 5 kDa appears to maximize their half-life time in bloodstream based on published data. Using PEG molecules with MW ≤ 2 kDa for coating does not help in prolonging blood circulation of GNPs regardless of their size. It is also important to keep in mind that nanoparticles with size <5 nm are quickly excreted *via* kidneys, usually within several hours.^21,39^ While there have been studies investigating the impact of GNP size and PEG MW on their blood circulation, to our knowledge this is the first systematic statistical analysis to establish the range of practical values for enhanced GNP design. In summary, this study lays the foundation for a data-driven approach to optimize GNP design for various biomedical applications, such as drug delivery and diagnostic imaging.

However, it is essential to acknowledge that blood circulation time is just one of the many factors that influence the overall performance of GNPs *in vivo*. Other factors, such as surface charge, shape, and targeting ligands, may also play crucial roles in determining the biodistribution, tumour uptake, and therapeutic efficacy of GNPs. Future studies should investigate the impact of these factors and their potential interactions with GNP size and PEG coating to develop a more comprehensive understanding of GNP behaviour in biological systems. Moreover, while this study focuses on data from mouse models, it is crucial to validate these findings in other animal models and, eventually, in clinical studies.

## Methods

### Literature search and data collection

We performed a comprehensive iterative literature search using PubMed, Web of Science, and Google Scholar and a combination of keywords including “gold nanoparticles”, “blood circulation”, “clearance”, “biodistribution” and “PEG”. We only selected original papers published in peer-reviewed journals, which resulted in an initial pool of ∼200 articles. These papers were further evaluated to select only (i) original studies which evaluated blood circulation of GNPs in mice after intravenous administration; (ii) studies using spherical GNPs with only PEG-based coating and no additional modification; (iii) studies that reported the size of GNPs, MW of PEG used in coating, and half-life of GNPs in mice blood after intravenous administration. Mice were selected as the model organism as initial screening of the literature showed that most of the biodistribution studies with GNPs were performed in mice. There were reports which used nanoparticles of other shapes which were not selected for the analysis as shape of the nanoparticles might have an influence on their cellular uptake and blood clearance.^29,40,41^ We also did not consider studies which used chemically modified derivatives of PEG (*i.e.*, addition of targeting moieties or polymers), or any other types of coatings to have a consistency for analysis. This selection process resulted in a final list of 12 papers which were used to extract a dataset of 41 combinations of reported numerical values of GNP diameter, MW of PEG, and half-life in blood after administration (Table S1 in the ESI†).^23,42–52^

### Statistical analysis

Data analysis was performed using GAM in R version 4.4.2., employing the ‘mgcv’ package version 1.9-1. The code used for the analysis can be found in the ESI.†

## Data availability

The data supporting this article and full code for analysis have been included as part of the ESI.†

## Author contributions

Dmitry Nevozhay: project conception, project design, data collection, data cleanup, statistical analysis, manuscript writing. Ronald Rauch: literature search and data collection. Zhongya Wang: statistical model validation. Konstantin V. Sokolov: project conception, project design, manuscript writing.

## Conflicts of interest

There are no conflicts to declare.

## Supplementary Material

NA-007-D4NA00782D-s001
