# Supplementary material for: Optimal size and PEG coating of gold nanoparticles for prolonged blood circulation: a statistical analysis of published data
Source: Nanoscale Adv. 2025 Jan 13;7(3):722–7. doi: 10.1039/d4na00782d (PMC11726233; doi:10.1039/d4na00782d)
Supplement: NA-007-D4NA00782D-s001 [file NA-007-D4NA00782D-s001.pdf]

Supplementary Information (SI) for Nanoscale Advances.
This journal is © The Royal Society of Chemistry 2024

Optimal size and PEG coating of gold nanoparticles for prolonged blood circulation: a statistical analysis of published data

Dmitry Nevozhay[1,*], Ronald Rauch[1], Zhongya Wang[2], and Konstantin V. Sokolov[1,3,4,]**

[1] Department of Imaging Physics, The University of Texas MD Anderson Cancer Center, Houston, TX 77030, USA

[2] Department of Biostatistics, The University of Texas MD Anderson Cancer Center, Houston, TX 77030, USA

[3] Department of Bioengineering, Rice University, Houston, TX 77005, USA

[4] Department of Biomedical Engineering, The University of Texas at Austin, Austin, TX 78712, USA

[*]Corresponding author: 1881 East Road, 3SCR2.3626, Houston, TX 77054, USA; e-mail: dnevozhay@mdanderson.org

[**]Corresponding author: 1881 East Road, 3SCR2.3606, Houston, TX 77054, USA; e-mail: ksokolov@mdanderson.org

N	GNP diameter, nm	PEG MW, kDa	Half-life time, h	Source
1	17.72	2	4	Perrault et al., 2009 [1]
2	17.72	5	29.7	Perrault et al., 2009 [1]
3	17.72	10	51.1	Perrault et al., 2009 [1]
4	31.28	2	2.4	Perrault et al., 2009 [1]
5	31.28	5	19.3	Perrault et al., 2009 [1]
6	31.28	10	22.7	Perrault et al., 2009 [1]
7	45.03	2	0.4	Perrault et al., 2009 [1]
8	45.03	5	14.1	Perrault et al., 2009 [1]
9	45.03	10	16.1	Perrault et al., 2009 [1]
10	66.54	2	1	Perrault et al., 2009 [1]
11	66.54	5	9.2	Perrault et al., 2009 [1]
12	66.54	10	11.3	Perrault et al., 2009 [1]
13	86.73	2	3.3	Perrault et al., 2009 [1]
14	86.73	10	6.6	Perrault et al., 2009 [1]
15	16.6	2	2.5	Perrault et al., 2009 [1]
16	22.6	2	4	Perrault et al., 2009 [1]
17	32.5	5	16.5	Perrault et al., 2009 [1]
18	43.3	10	11.6	Perrault et al., 2009 [1]
19	83.5	10	7.2	Perrault et al., 2009 [1]
20	43.6	5	8.19	You et al., 2014 [2]
21	15	5	31.9	Chou et al., 2012 [3]
22	100	5	7.3	Chou et al., 2012 [3]
23	2	0.2	5	Arvizo et al., 2011 [4]
24	25	5	20	Ashton et al., 2018 [5]
25	20	5	22.5	Zhang et al., 2009 [6]
26	40	5	10.1	Zhang et al., 2009 [6]
27	80	5	15.8	Zhang et al., 2009 [6]
28	16	2	6.65	Liu et al., 2014 [7]
29	40	4.4	6	Rehor et al., 2008 [8]
30	100	4.4	2.9	Rehor et al., 2008 [8]
31	10	2	14.57	Cai et al., 2007 [9]
32	4.3	0.35	0.87	Zhao et al., 2014 [10]
33	6.9	1	2.62	Zhao et al., 2014 [10]
34	13	5	28.5	Cho et al., 2009 [11]
35	5	5	48.9	Choi et al., 2011 [12]
36	22	5	31.8	Choi et al., 2011 [12]
37	41	4.1	13.8	Choi et al., 2011 [12]
38	51	5	13.7	Choi et al., 2011 [12]
39	58	7.3	11.4	Choi et al., 2011 [12]
40	77	10	8.7	Choi et al., 2011 [12]
41	98	20	6.8	Choi et al., 2011 [12]

Table S1. Data points used in the statistical analysis and their published sources.

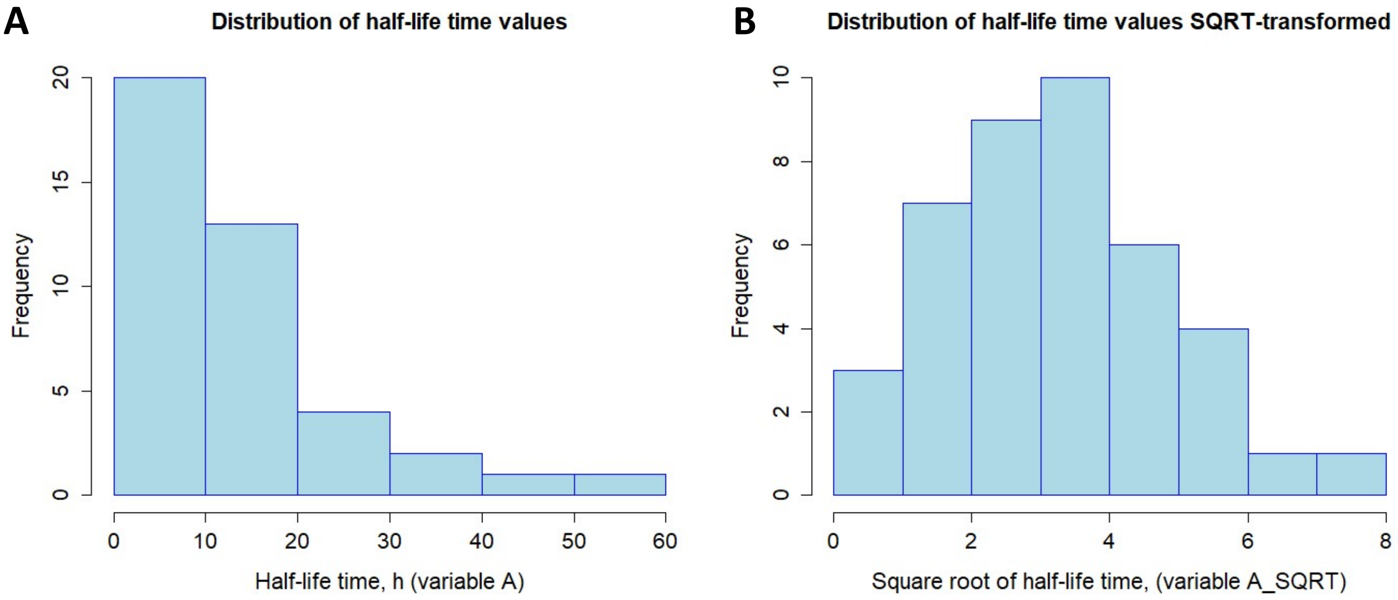

Figure S1. Distribution of original (A) and square root transformed (B) half-life time values.

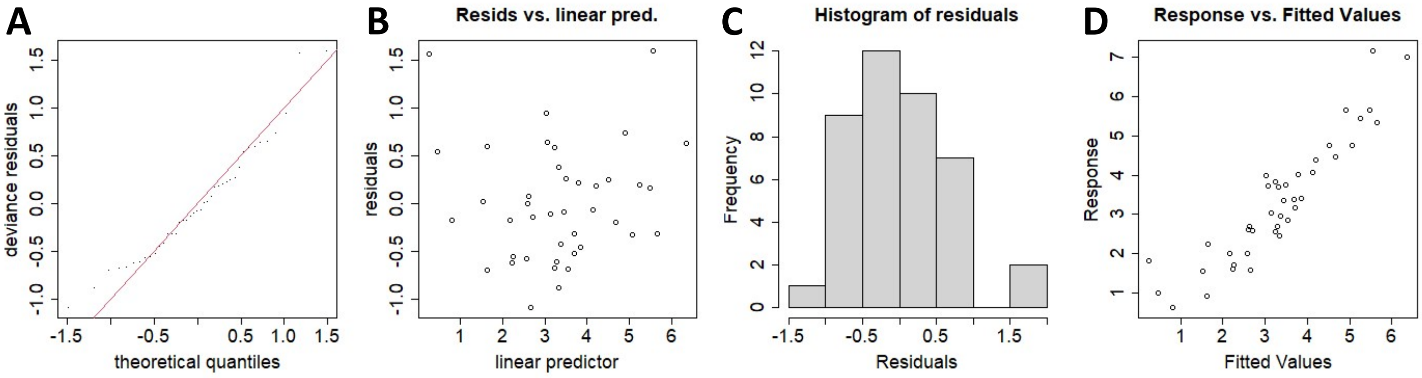

Figure S2. Diagnostic plots for model without interaction. (A) Q-Q plot, (B) plot of residuals versus predictors, (C) distribution of residuals, (D) plot of response against fitted values.

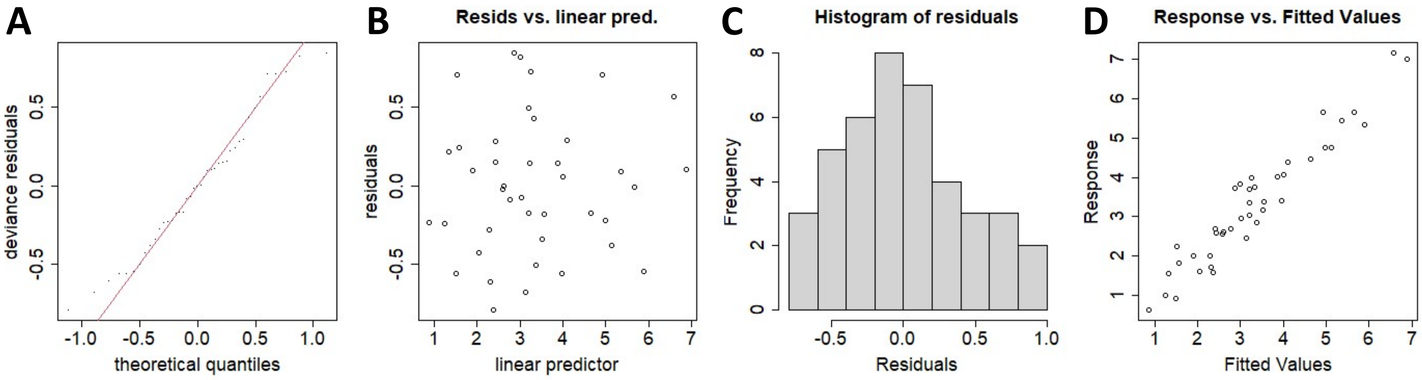

Figure S3. Diagnostic for model with interaction. (A) Q-Q plot, (B) plot of residuals versus predictors, (C) distribution of residuals, (D) plot of response against fitted values.

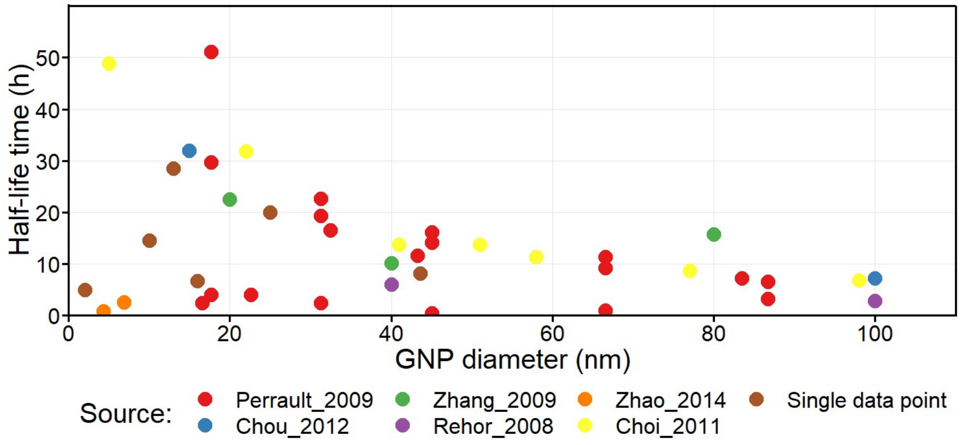

Figure S4. Data points used in the study are grouped by their published source. The 'Single data point' category represents six papers, each containing a single data point.

Weighted Generalized Additive Model (GAM) analysis

To address potential bias from overrepresentation of 5 kDa PEG MW data (39% of the dataset), we performed additional analysis using weighted GAM. This approach assigns inverse frequency weights to each PEG MW category, effectively reducing the influence of overrepresented data points while preserving the original dataset. The detailed code for this analysis is included at the end of this Supporting Information file. Inverse frequency weights were computed for each PEG MW value using the formula

$$PEG\ MW\ Weight = \frac{1}{Frequency\ of\ PEG\ MW\ value}$$

These weights were applied using the weights argument in the GAM function. The model maintained the same structure as the original unweighted model (A_sqrt ~ s(B) + s(C) + ti(B, C)) and used square root-transformed half-life values as in the main text.

Diagnostic plots demonstrated good fit quality of weighted model and residual behaviour consistent with the original model. The weighted model showed strong performance ($R^2 = 0.81$ vs 0.90 in original model, Deviance explained = 85.8% vs 92.3%). All smooth terms remained highly significant (GNP size: $p < 0.001$; PEG MW: $p < 0.001$; Interaction: $p = 0.001$), consistent with the original model. Similar to the unweighted model, GNP size showed an inverse relationship with half-life time below 40nm, and PEG MW demonstrated a threshold effect around 5 kDa (Figure S5A, B). Interaction patterns remained stable and also consistent with unweighted model (Figure S5C).

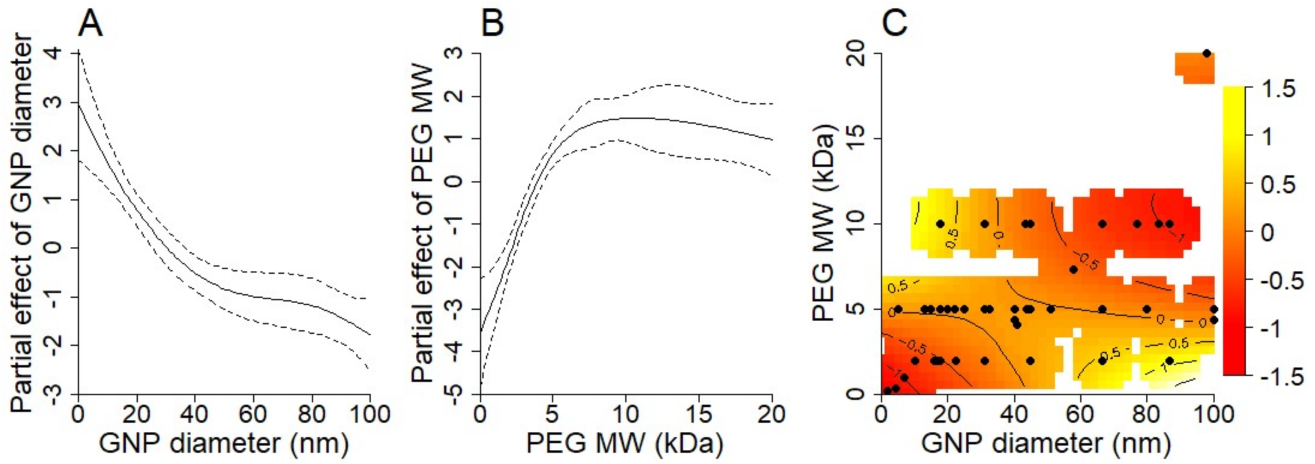

Figure S5. Results from weighted GAM analysis. Main effects (A) GNP diameter, (B) PEG MW; (C) Interaction term between GNP size and PEG MW. Dashed lines represent confidence intervals in A, B and dark dots represent data points in C.

The weighted GAM analysis strongly supports the robustness of our findings. Despite deliberately reducing the influence of overrepresented PEG MW values, the model identifies the same key relationships and optimal parameter ranges. The similarity in patterns between weighted and unweighted analyses, combined with good performance metrics in both models, suggests our conclusions reflect genuine underlying relationships rather than artifacts of data imbalance.

The slightly lower R^2 and deviance explained values in the weighted model are expected, as reducing the influence of numerous 5 kDa data points increases relative variability. However, the maintenance of significant effects and consistent patterns provides strong evidence that our conclusions about optimal GNP size and PEG MW combinations are reliable, independent of data distribution biases.

Figure S5 shows the partial effects and interaction terms of the weighted model (B), which can be compared to the original unweighted analysis from Figure 2 in the main text. The consistency in patterns, particularly around key thresholds (40 nm for GNP size, 5 kDa for PEG MW), provides visual confirmation that our conclusions are robust to potential bias from data imbalance."

Sensitivity analysis using bootstrapping.

Sensitivity analysis using bootstrapping techniques is a statistical approach used to evaluate the robustness of conclusions by examining how changes in data distribution or model assumptions affect the results. This method involves resampling the data with replacement to create random datasets using original data. In this analysis, we generated 1,000 bootstrapped datasets, each with potentially different emphases on certain PEG molecular weight intervals due to the random sampling process. For each bootstrap sample, we refitted the model and recalculated the key metrics, including the relationship between PEG molecular weight and GNP size.

Next, we constructed a "reconstructed” model by averaging the coefficients obtained from each bootstrapped dataset. We applied both the original model (constructed with the original dataset size of 41) and the reconstructed model to predict the outcomes using the original data. A comparison of predictions from the two models, shown in Figure S6, reveals that the predictions are randomly distributed around the diagonal line, indicating that the model is stable and does not seem to be significantly influenced by data imbalance.

The bootstrap analysis provides strong evidence for the robustness of our statistical model and its conclusions. While it doesn't correct for data imbalance, it demonstrates that our findings about optimal GNP size and PEG MW combinations are stable and reproducible across different random samples of the available data. The consistency between original and reconstructed model predictions suggests that our conclusions are not artifacts of particular data points but reflect genuine underlying relationships in GNP circulation behavior.

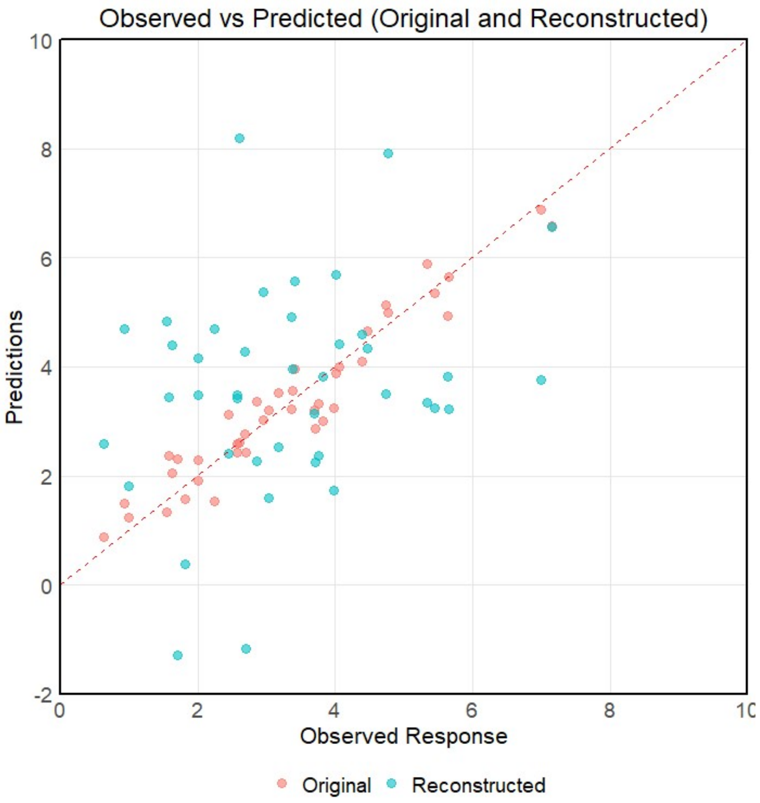

Figure S6. Comparison of predictions from original and bootstrapped GAM models. The plot shows the relationship between observed half-life values (square-root transformed) and values predicted by statistical models. Pink points represent predictions from the original GAM model (Model 2, with interaction term from the main text), while cyan points show predictions from the reconstructed model based on 1,000 bootstrap iterations. The dashed red line represents the ideal 1:1 relationship between observed and predicted values. The reconstructed predictions appear to show more scatter than the original predictions, which is expected due to the averaging of bootstrap iterations. The random scattering and alignment of both sets of predictions along the diagonal line suggests model stability and robustness to data sampling variations.

R code used for visualization and analysis:

```
# Load the necessary packages
library(ggplot2)
library(gridExtra)
library(mgcv)
library(RColorBrewer)
library(fields)

# Define the data as a string
data_string <- "17.72	2	4	Perrault_2009
17.72	5	29.7	Perrault_2009
17.72	10	51.1	Perrault_2009
31.28	2	2.4	Perrault_2009
31.28	5	19.3	Perrault_2009
31.28	10	22.7	Perrault_2009
45.03	2	0.4	Perrault_2009
45.03	5	14.1	Perrault_2009
45.03	10	16.1	Perrault_2009
66.54	2	1	Perrault_2009
66.54	5	9.2	Perrault_2009
66.54	10	11.3	Perrault_2009
86.73	2	3.3	Perrault_2009
86.73	10	6.6	Perrault_2009
16.6	2	2.5	Perrault_2009
22.6	2	4	Perrault_2009
32.5	5	16.5	Perrault_2009
43.3	10	11.6	Perrault_2009
83.5	10	7.2	Perrault_2009
43.6	5	8.19	You_2014
15	5	31.9	Chou_2012
100	5	7.3	Chou_2012
2	0.2	5	Arvisio_2011
25	5	20	Ashton_2018
20	5	22.5	Zhang_2009
40	5	10.1	Zhang_2009
80	5	15.8	Zhang_2009
16	2	6.65	Liu_2014
40	4.4	6	Rehor_2008
100	4.4	2.9	Rehor_2008
10	2	14.57	Cai_2007
4.3	0.35	0.87	Zhao_2014
6.9	1	2.62	Zhao_2014
13	5	28.5	Cho_2009
5	5	48.9	Choi_2011
22	5	31.8	Choi_2011
41	4.1	13.8	Choi_2011
51	5	13.7	Choi_2011
58	7.3	11.4	Choi_2011
77	10	8.7	Choi_2011
98	20	6.8	Choi_2011"

# Read the string into a dataframe
df <- read.table(text=data_string, header=FALSE, sep="\t")

# Rename the columns
colnames(df) <- c("B", "C", "A", "Source")

# plotting A and square root transformed A to assess their distributions
df$A_sqrt <- sqrt(df$A)
par(mfrow = c(1, 2))
hist(df$A, breaks=6, main="Distribution of half-life time values", xlab="Half-life time, h
(variable A)", col="lightblue", border="blue", cex.main=1.4,cex.lab=1.4, cex.axis=1.3)
hist(df$A_sqrt, breaks=6, main="Distribution of half-life time values SQRT-transformed",
xlab="Square root of half-life time, (variable A_SQRT)", col="lightblue", border="blue",
cex.main=1.4,cex.lab=1.4, cex.axis=1.3)

# Fit the GAM (no interaction) with SQRT normalized A and display summary
model_sqrt <- gam(A_sqrt ~ s(B) + s(C), data = df, method = "REML")
summary(model_sqrt)
```

```
# Fit the GAM (interaction) with SQRT normalized A
model_sqrt_int <- gam(A_sqrt ~ s(B) + s(C) + ti (B, C), data = df, method = "REML")
summary(model_sqrt_int)

# Compare two model using ANOVA
anova(model_sqrt, model_sqrt_int, test="Chisq")

# Estimating AIC and BIC for both models
AIC(model_sqrt, model_sqrt_int)
BIC(model_sqrt, model_sqrt_int)

# Displaying diagnostic plots for models
old_par <- par()
par(mfrow = c(1, 4), cex.axis=1.7, cex.lab=1.7, cex.main=1.7)
gam.check(model_sqrt)
gam.check(model_sqrt_int)
par(old_par)

xlims <- c(0, 100)
ylims <- c(0, 20)

xmin <- 0
xmax <- max(df$B)
ymin <- 0
ymax <- max(df$A)

# Displaying main affects and interaction terms for the model

minZ <- -1.5
maxZ <- 1.5

# Create a custom gradient
breaks <- c(seq(minZ, -1, length.out = 51),
            seq(-1, 1, length.out = 51)[-1],
            seq(1, maxZ, length.out = 51)[-1])
cols <- c(rep("red", 50), colorRampPalette(c("red", "yellow"))(50), rep("yellow", 50))

# Define a layout for the plots
layout(matrix(c(1, 2, 3), 1, 3, byrow = TRUE), widths = c(1, 1, 1.1), heights = 1)

# Set margins and outer margins
par(oma = c(0, 0, 0, 5), mar = c(5, 5, 4, 2) + 0.1, xpd = NA)

### Plot A ###
plot(model_sqrt_int, select = 1, scheme = 2, shade = FALSE, se = TRUE, rug = FALSE,
     xlim = c(0, 100), ylim = c(-3, 4), xaxs = "i", yaxs = "i",
     xlab = "", ylab = "", main = "", axes = FALSE)
axis(1, at = seq(0, 100, 20), cex.axis = 2)
axis(2, at = seq(-3, 4, 1), pos = 0, cex.axis = 2)
mtext("GNP diameter (nm)", side = 1, line = 3, cex = 1.5)
mtext("Partial effect of GNP diameter", side = 2, line = 3, cex = 1.5)
mtext("A", side = 3, line = 1, adj = 0, cex = 2)

### Plot B ###
plot(model_sqrt_int, select = 2, scheme = 2, shade = FALSE, se = TRUE, rug = FALSE,
     xlim = c(0, 20), ylim = c(-5, 3), xaxs = "i", yaxs = "i",
     xlab = "", ylab = "", main = "", axes = FALSE)
axis(1, at = seq(0, 20, 5), cex.axis = 2)
axis(2, at = seq(-5, 3, 1), pos = 0, cex.axis = 2)
mtext("PEG MW (kDa)", side = 1, line = 3, cex = 1.5)
mtext("Partial effect of PEG MW", side = 2, line = 3, cex = 1.5)
mtext("B", side = 3, line = 1, adj = 0, cex = 2)

### Plot C ###
plot(model_sqrt_int, select = 3, scheme = 2, shade = FALSE, se = FALSE, rug = FALSE,
    xlim = c(0, 100), ylim = c(0, 20), xaxs = "i", yaxs = "i",
     xlab = "", ylab = "", main = "", axes = FALSE,
     contour.col = "black", labcex = 0.7, lwd = 1 )
```

```
axis(1, at = seq(0, 100, 20), cex.axis = 2)
axis(2, at = seq(0, 20, 5), cex.axis = 2)
mtext("GNP diameter (nm)", side = 1, line = 3, cex = 1.5)
mtext("PEG MW (kDa)", side = 2, line = 3, cex = 1.5)
mtext("C", side = 3, line = 1, adj = 0, cex = 2)

# Add points on top of Plot C
par(new = TRUE)
plot(df$B, df$C, pch = 16, cex = 1.5,
     xlim = c(0, 100), ylim = c(0, 20),
     xlab = "", ylab = "", axes = FALSE, xaxs = "i", yaxs = "i")

### Add Vertical Color Legend ###
par(xpd = NA)
image.plot(legend.only = TRUE,
           zlim = c(-1.5, 1.5),
           col = cols,
           breaks = breaks,  # Use the same breaks as in the main plot
           horizontal = FALSE,
           legend.width = 6,
           axis.args = list(
             at = seq(-1.5, 1.5, 0.5),
             labels = seq(-1.5, 1.5, 0.5),
             cex.axis = 2
           ),
           add = TRUE,
           smallplot = c(0.95, 1, 0.2, 0.8))

# Plotting data with rainbow colors
# Creating a custom color palette
colors <- c(rainbow(6))
stops <- c(0, 0.125, 0.25, 0.375, 0.5, 1)

# Creating the plot with custom color gradient
plot <- ggplot(df, aes(x = B, y = A, color = C)) +
  # Add grid lines first (to be underneath points)
  geom_vline(xintercept = seq(0, 110, by = 20), color = "grey90", linewidth = 0.5) +
  geom_hline(yintercept = seq(0, 60, by = 10), color = "grey90", linewidth = 0.5) +
  geom_point(size = 7) +
  scale_color_gradientn(colors = colors, values = stops) +
  # Set proper axis limits with new x-axis breaks
  scale_x_continuous(limits = c(0, 110), breaks = seq(0, 100, by = 20), expand = c(0, 0)) +
  scale_y_continuous(limits = c(0, 60), breaks = seq(0, 50, by = 10), expand = c(0, 0)) +
  coord_cartesian(xlim = c(-0.2, 110.1), ylim = c(0, 60.3), clip = "on") +
  theme_minimal() +
  theme(
    text = element_text(size = 32),
    legend.position = "bottom",
    legend.key.width = unit(2, "cm"),
    panel.grid = element_blank(),
    axis.line = element_blank(),
    axis.ticks = element_line(color = "black", linewidth = 1.2),
    axis.ticks.length = unit(0.25, "cm"),
    axis.text = element_text(color = "black"),
    axis.title = element_text(color = "black")
  ) +
  # Draw x-axis first
  geom_segment(
    data = data.frame(x = 0),
    aes(x = 0, xend = 110, y = 0, yend = 0),
    color = "black",
    linewidth = 1.2,
    inherit.aes = FALSE
  ) +
  # Draw y-axis second (on top)
  geom_segment(
    data = data.frame(y = 0),
    aes(x = 0, xend = 0, y = 0, yend = 60),
    color = "black",
    linewidth = 1.2,
```

```
    inherit.aes = FALSE
  ) +
  geom_segment(
    x = 0, xend = 110, y = 60, yend = 60,
    color = "black", linewidth = 1.2, inherit.aes = FALSE
  ) +
  # Draw the right line
  geom_segment(
    x = 110, xend = 110, y = 0, yend = 60,
    color = "black", linewidth = 1.2, inherit.aes = FALSE
  ) +
  labs(x = "GNP diameter (nm)",
       y = "Half-life time (h)",
       color = "PEG MW (kDa)")
plot

# Exporting TOC
png("toc_graphic.png",
    width = 1890,
    height = 942,
    type = "cairo",    # Sometimes helps with rendering
    units = "px",
    res = 96)          # Standard screen resolution
print(plot)
dev.off()

# Plotting data by the source
# Filter out sources with only one data point
source_count <- table(df$Source)
valid_sources <- names(source_count[source_count > 1])
single_sources <- names(source_count[source_count <= 1])
df_filtered <- df[df$Source %in% valid_sources, ]
df_single_point <- df[df$Source %in% single_sources, ]
df_single_point$Source <- "Single data point"
combined_df <- rbind(df_filtered, df_single_point)
combined_df$Source <- factor(combined_df$Source, levels = unique(combined_df$Source))

# Choosing a palette with 7 distinct colors
color_palette <- brewer.pal(7, "Set1")

plot <- ggplot(combined_df, aes(x = B, y = A, color = factor(Source))) +
  # Grid lines underneath points
  geom_vline(xintercept = seq(xmin, xmax, by = 20), color = "grey90", linewidth = 0.5) +
  geom_hline(yintercept = seq(ymin, ymax, by = 10), color = "grey90", linewidth = 0.5) +
  # Points
  geom_point(size = 7) +
  # Manual color palette
  scale_color_manual(values = color_palette) +
  # Proper axis limits and zero-expansion
  scale_x_continuous(limits = c(xmin, xmax), breaks = seq(xmin, xmax, by = 20), expand = c(0, 0))
+
  scale_y_continuous(limits = c(ymin, ymax), breaks = seq(ymin, ymax, by = 10), expand = c(0, 0))
+
  coord_cartesian(xlim = c(0, 110.2), ylim = c(0, 60.3), clip = "on") +
  theme_minimal() +
  theme(
    text = element_text(size = 30),
    legend.position = "bottom",
    legend.key.width = unit(2, "cm"),
    panel.grid = element_blank(),
    # Enable axis lines
    axis.line = element_line(color = "black", linewidth = 1.2),
    axis.ticks = element_line(color = "black", linewidth = 1.2),
    axis.ticks.length = unit(0.25, "cm"),
    axis.text = element_text(color = "black"),
    axis.title = element_text(color = "black")
  ) +
  # Draw the x-axis line
  geom_segment(
    x = 0, xend = 110, y = 0, yend = 0,
```

```
    color = "black", linewidth = 1.2, inherit.aes = FALSE
  ) +
  # Draw the y-axis line
  geom_segment(
    x = 0, xend = 0, y = 0, yend = 60,
    color = "black", linewidth = 1.2, inherit.aes = FALSE
  ) +
  geom_segment(
    x = 0, xend = 110, y = 60, yend = 60,
    color = "black", linewidth = 1.2, inherit.aes = FALSE
  ) +
  # Draw the right line
  geom_segment(
    x = 110, xend = 110, y = 0, yend = 60,
    color = "black", linewidth = 1.2, inherit.aes = FALSE
  ) +
  labs(
    x = "GNP diameter (nm)",
    y = "Half-life time (h)",
    color = "Source:"
  )
plot

#### Weighted GAM analysis

# Calculate Inverse Frequency Weights
peg_counts <- table(df$C)
df$Weight <- 1 / peg_counts[as.character(df$C)]

model_weighted_int <- gam(A_sqrt ~ s(B) + s(C) + ti(B, C), data=df, weights=df$Weight,
method="REML")

# Compare Models
summary(model_weighted_int)
anova(model_sqrt_int, model_weighted_int, test="Chisq")

# Evaluate Model Fit with AIC and BIC
AIC(model_sqrt_int, model_weighted_int)
BIC(model_sqrt_int, model_weighted_int)

# Displaying diagnostic plots for models
old_par <- par()
par(mfrow = c(1, 4), cex.axis=1.7, cex.lab=1.7, cex.main=1.7)
gam.check(model_sqrt_int)
gam.check(model_weighted_int)
par(old_par)

# Displaying main affects and interaction terms for the model
minZ <- -1.5
maxZ <- 1.5

# Create a custom gradient
breaks <- c(seq(minZ, -1, length.out = 51),
            seq(-1, 1, length.out = 51)[-1],
            seq(1, maxZ, length.out = 51)[-1])
cols <- c(rep("red", 50), colorRampPalette(c("red", "yellow"))(50), rep("yellow", 50))

# Define a layout for the plots
layout(matrix(c(1, 2, 3), 1, 3, byrow = TRUE), widths = c(1, 1, 1.1), heights = 1)

# Set margins and outer margins
par(oma = c(0, 0, 0, 5), mar = c(5, 5, 4, 2) + 0.1, xpd = NA)

### Plot A ###
plot(model_weighted_int, select = 1, scheme = 2, shade = FALSE, se = TRUE, rug = FALSE,
     xlim = c(0, 100), ylim = c(-3, 4), xaxs = "i", yaxs = "i",
     xlab = "", ylab = "", main = "", axes = FALSE)
axis(1, at = seq(0, 100, 20), cex.axis = 2)
axis(2, at = seq(-3, 4, 1), pos = 0, cex.axis = 2)
mtext("GNP diameter (nm)", side = 1, line = 3, cex = 1.5)
```

```
mtext("Partial effect of GNP diameter", side = 2, line = 3, cex = 1.5)
mtext("A", side = 3, line = 1, adj = 0, cex = 2)

### Plot B ###
plot(model_weighted_int, select = 2, scheme = 2, shade = FALSE, se = TRUE, rug = FALSE,
     xlim = c(0, 20), ylim = c(-5, 3), xaxs = "i", yaxs = "i",
     xlab = "", ylab = "", main = "", axes = FALSE)
axis(1, at = seq(0, 20, 5), cex.axis = 2)
axis(2, at = seq(-5, 3, 1), pos = 0, cex.axis = 2)
mtext("PEG MW (kDa)", side = 1, line = 3, cex = 1.5)
mtext("Partial effect of PEG MW", side = 2, line = 3, cex = 1.5)
mtext("B", side = 3, line = 1, adj = 0, cex = 2)

### Plot C ###
plot(model_weighted_int, select = 3, scheme = 2, shade = FALSE, se = FALSE, rug = FALSE,
     xlim = c(0, 100), ylim = c(0, 20), xaxs = "i", yaxs = "i",
     xlab = "", ylab = "", main = "", axes = FALSE,
     contour.col = "black", labcex = 0.7, lwd = 1 )

axis(1, at = seq(0, 100, 20), cex.axis = 2)
axis(2, at = seq(0, 20, 5), cex.axis = 2)
mtext("GNP diameter (nm)", side = 1, line = 3, cex = 1.5)
mtext("PEG MW (kDa)", side = 2, line = 3, cex = 1.5)
mtext("C", side = 3, line = 1, adj = 0, cex = 2)

# Add points on top of Plot C
par(new = TRUE)
plot(df$B, df$C, pch = 16, cex = 1.5,
     xlim = c(0, 100), ylim = c(0, 20),
     xlab = "", ylab = "", axes = FALSE, xaxs = "i", yaxs = "i")

### Add Vertical Color Legend ###
par(xpd = NA)
image.plot(legend.only = TRUE,
           zlim = c(-1.5, 1.5),
           col = cols,
           breaks = breaks,  # Use the same breaks as in the main plot
           horizontal = FALSE,
           legend.width = 6,
           axis.args = list(
             at = seq(-1.5, 1.5, 0.5),
             labels = seq(-1.5, 1.5, 0.5),
             cex.axis = 2
           ),
           add = TRUE,
           smallplot = c(0.95, 1, 0.2, 0.8))

#### Sensitivity analysis by bootstrapping

library(boot)

expected_coef_length<-31
model_function <- function(data, indices) {
  boot_data <- data[indices, ]

  # Fit the model with error handling
  model <- gam(A_sqrt ~ s(B, k = 5) + s(C, k = 4) + ti(B, C, k = c(4, 4)),
               data = boot_data, method = "REML")
  coefs <- coef(model)

  # Ensure consistent length by padding with NA if needed
  full_coefs <- rep(NA, expected_coef_length)  # Replace with actual number of coefficients
  full_coefs[seq_along(coefs)] <- coefs
  return(full_coefs)
}

set.seed(1234)  # For reproducibility
bootstrap_results <- boot(data = df, statistic = model_function, R = 1000)
coefs <- apply(bootstrap_results$t, 2, function(x) {
  mean(ifelse(is.na(x), 0, x))
```

```
})

model_int <- gam(A_sqrt ~ s(B) + s(C) + ti(B, C), data =df, method = "REML")
X <- predict(model_int, newdata = df, type = "lpmatrix")

# Reconstruct predictions
# Multiply the design matrix (X) by the coefficients
reconstructed_preds <- X %*% coefs

# Add the reconstructed predictions to the dataset
df$reconstructed_preds <- reconstructed_preds

# Compare reconstructed predictions with fitted values
comparison<-data.frame(
  Original_Predictions = predict(model_int),
  Reconstructed_Predictions = reconstructed_preds
)

# Compute Difference
comparison$Difference = comparison$Original_Predictions - comparison$Reconstructed_Predictions

# Print Comparison Table
head(comparison)

# Compare Predictions vs Observed Response
comparison$Observed_Response = df$A_sqrt

# Plot for Visual Comparison (Observed vs Predicted)
ggplot(comparison, aes(x = Observed_Response)) +
  geom_point(aes(y = Original_Predictions, color = "Original"),
             alpha = 0.6, size = 3) +
  geom_point(aes(y = Reconstructed_Predictions, color = "Reconstructed"),
             alpha = 0.6, size = 3) +
  geom_abline(slope = 1, intercept = 0, linetype = "dashed", color = "red") +
  labs(
    title = "Observed vs Predicted (Original and Reconstructed)",
    x = "Observed Response",
    y = "Predictions",
    color = ""
  ) +
  scale_y_continuous(
    limits = c(-2, 10),
    breaks = seq(-2, 10, by = 2),
    expand = c(0, 0),  # Remove padding
    position = "left"  # Ensure axis is on the left
  ) +
  scale_x_continuous(
    limits = c(0, 10),
    breaks = seq(0, 10, by = 2),
    expand = c(0, 0),  # Remove padding
    position = "bottom"  # Ensure axis is on the bottom
  ) +
  theme_minimal() +
  theme(
    text = element_text(size = 14),
    axis.title = element_text(size = 16),
    axis.text = element_text(size = 14),
    plot.title = element_text(size = 18, hjust = 0.5),
    legend.position = "bottom",
    legend.text = element_text(size = 14),
    legend.title = element_text(size = 16),
    panel.border = element_rect(color = "black", fill = NA, linewidth = 1),
    # Add specific axis line styling
    axis.line = element_line(color = "black"),
    # Move axis lines to cross at (0, -2)
    axis.line.x = element_line(color = "black", size = 0.5,
                               linetype = "solid"),
    axis.line.y = element_line(color = "black", size = 0.5,
                               linetype = "solid"),
    panel.grid.major = element_line(color = "grey90"),
```

```
    panel.grid.minor = element_blank()
  ) +
  coord_cartesian(clip = "off")  # Prevent clipping of axis lines
```

References

1. Perrault, S. D.; Walkey, C.; Jennings, T.; Fischer, H. C.; Chan, W. C., Mediating tumor targeting efficiency of nanoparticles through design. *Nano Lett* **2009,** *9* (5), 1909-15.
2. You, J.; Zhou, J.; Zhou, M.; Liu, Y.; Robertson, J. D.; Liang, D.; Van Pelt, C.; Li, C., Pharmacokinetics, clearance, and biosafety of polyethylene glycol-coated hollow gold nanospheres. *Part Fibre Toxicol* **2014,** *11*, 26.
3. Chou, L. Y.; Chan, W. C., Fluorescence-tagged gold nanoparticles for rapidly characterizing the size-dependent biodistribution in tumor models. *Adv Healthc Mater* **2012,** *1* (6), 714-21.
4. Arvizo, R. R.; Miranda, O. R.; Moyano, D. F.; Walden, C. A.; Giri, K.; Bhattacharya, R.; Robertson, J. D.; Rotello, V. M.; Reid, J. M.; Mukherjee, P., Modulating pharmacokinetics, tumor uptake and biodistribution by engineered nanoparticles. *PLoS One* **2011,** *6* (9), e24374.
5. Ashton, J. R.; Gottlin, E. B.; Patz, E. F., Jr.; West, J. L.; Badea, C. T., A comparative analysis of EGFR-targeting antibodies for gold nanoparticle CT imaging of lung cancer. *PLoS One* **2018,** *13* (11), e0206950.
6. Zhang, G.; Yang, Z.; Lu, W.; Zhang, R.; Huang, Q.; Tian, M.; Li, L.; Liang, D.; Li, C., Influence of anchoring ligands and particle size on the colloidal stability and in vivo biodistribution of polyethylene glycol-coated gold nanoparticles in tumor-xenografted mice. *Biomaterials* **2009,** *30* (10), 1928-36.
7. Liu, X.; Li, H.; Chen, Y.; Jin, Q.; Ren, K.; Ji, J., Mixed-charge nanoparticles for long circulation, low reticuloendothelial system clearance, and high tumor accumulation. *Adv Healthc Mater* **2014,** *3* (9), 1439-47.
8. Rehor, A.; Schmoekel, H.; Tirelli, N.; Hubbell, J. A., Functionalization of polysulfide nanoparticles and their performance as circulating carriers. *Biomaterials* **2008,** *29* (12), 1958-66.
9. Cai, Q. Y.; Kim, S. H.; Choi, K. S.; Kim, S. Y.; Byun, S. J.; Kim, K. W.; Park, S. H.; Juhng, S. K.; Yoon, K. H., Colloidal gold nanoparticles as a blood-pool contrast agent for X-ray computed tomography in mice. *Invest Radiol* **2007,** *42* (12), 797-806.
10. Zhao, Y.; Sultan, D.; Detering, L.; Luehmann, H.; Liu, Y., Facile synthesis, pharmacokinetic and systemic clearance evaluation, and positron emission tomography cancer imaging of (6)(4)Cu-Au alloy nanoclusters. *Nanoscale* **2014,** *6* (22), 13501-9.
11. Cho, W. S.; Cho, M.; Jeong, J.; Choi, M.; Cho, H. Y.; Han, B. S.; Kim, S. H.; Kim, H. O.; Lim, Y. T.; Chung, B. H.; Jeong, J., Acute toxicity and pharmacokinetics of 13 nm-sized PEG-coated gold nanoparticles. *Toxicol Appl Pharmacol* **2009,** *236* (1), 16-24.
12. Choi, C. H.; Zuckerman, J. E.; Webster, P.; Davis, M. E., Targeting kidney mesangium by nanoparticles of defined size. *Proc Natl Acad Sci U S A* **2011,** *108* (16), 6656-61.
